# Efficacy and safety of *Rhodiola crenulata* extract in the treatment of acute high altitude disease, based on studies involving populations in China: A systematic review and meta-analysis

**DOI:** 10.3389/fphar.2025.1595953

**Published:** 2025-06-13

**Authors:** Zixuan Gao, Yaoyuan Liu, Weiwen Liao, Wenting Song, Xinyi Zhang, Hanqing Lin, Han Zhang, Tao Zhang, Wentai Pang

**Affiliations:** ^1^ School of Traditional Chinese Medicine, Tianjin University of Traditional Chinese Medicine, Tianjin, China; ^2^ First Teaching Hospital of Tianjin University of Traditional Chinese Medicine, National Clinical Research Center for Chinese Medicine Acupuncture and Moxibustion, Tianjin, China

**Keywords:** Rhodiola rosea, roseroot, altitude sickness, acute mountain sickness, AMS

## Abstract

**Introduction:**

To evaluate the efficacy and safety of *Rhodiola crenulata* extract (RCE) for the treatment of patients with acute high altitude disease (AHAD).

**Methods:**

This study systematically retrieved randomized controlled trials (RCTs) published prior to September 2024 from eight distinct databases. It included AHAD patients, with the control group receiving either conventional western medicine (WM) or placebo, and the experimental group receiving RCE alone or in conjunction with WM. The primary outcomes were arterial oxygen saturation (SaO_2_) and arterial partial pressure of oxygen (PaO_2_). The secondary outcomes were total clinical efficacy, systolic blood pressure (SBP), diastolic blood pressure (DBP) and heart rate (HR). Adverse events incidence was analyzed to assess safety. The meta-analysis was performed with Review Manager 5.4, and the evidence’s certainty was assessed using the GRADE approach.

**Results:**

This study included 19 eligible RCTs with 1,690 participants. In improving SaO_2_, PaO_2_ and total clinical efficacy, no significant differences were found between RCE and WM, but RCE was more effective than placebo. RCE showed no significant effect in reducing SBP, DBP and HR. Regarding safety, the experimental group demonstrated superior performance compared to the control group.

**Conclusion:**

RCE may enhance blood oxygen levels and mitigate clinical symptoms in the treatment of AHAD with favorable safety. Nonetheless, it is imperative to undertake further rigorous RCTs to validate these findings.

**Systematic Review Registration:**

https://www.crd.york.ac.uk/PROSPERO/myprospero, identifier CRD42024593081.

## 1 Introduction

High altitude disease (HAD) is an idiopathic disease that occurs in high-altitude regions, with hypoxia being the primary cause (Bärtsch and Gibbs, 2007; Wu, 2014; Zhaxi et al., 2024). Acute high-altitude disease (AHAD) can occur with initial or rapid exposure to high altitudes, and in serious cases, it may result in pulmonary and cerebral edema, which could be life-threatening (Pena et al., 2022). AHAD is a syndrome primarily marked by headache, along with symptoms like nausea, fatigue, dyspnea, insomnia, and dizziness (Wu et al., 2018). In 2000, over 100 million people traveled to high-altitude areas, a trend that continues to grow, especially in regions like the Qinghai-Tibet Plateau (Faulhaber et al., 2014; Ma et al., 2022; Zhaxi et al., 2024). AHAD typically appears within 6 h of ascending above 2,500 m, peaking within 12–96 h. It affects over 25% of those reaching 3,500 m and more than 50% at elevations above 6,000 m, indicating a significant impact on a significant portion of the population (Wu et al., 2018; Guo et al., 2024).

Currently, the conventional treatment drugs for AHAD include acetazolamide, dexamethasone, aminophylline, etc (Imray et al., 2010; Hung et al., 2019). They exert effects that enhance anti-hypoxia capacity, increase blood flow, and improve acid-base balance (Ren and Wang, 2011). Although these medications act rapidly, they have significant side effects and are primarily used for emergency situations in AHAD (Ren and Wang, 2011). Given the limitations of current pharmacological approaches, attention has turned toward traditional herbal remedies with historical usage in high-altitude regions, such as *Rhodiola crenulata*.

*Rhodiola crenulata* (Hook.f. and Thomson) H.Ohba (World Flora Online, 1976), a traditional Chinese medicinal plant, thrives at 3,000-4,000 m and is used in Tibet to fight fatigue and adapt to high altitudes (Ren, 2022). At present, extracts derived from *R. crenulata* have been incorporated into a variety of Chinese patent medicine, and *R. crenulata* extract (RCE) has become a leading remedy in China for preventing and treating altitude sickness (Chen, 2013; Feng et al., 2013; Tang et al., 2015; Li et al., 2023). Research showed that RCE contained various bioactive compounds, including salidroside and gallic acid. It offers immunomodulatory, antioxidant, anti-inflammatory, and neuroprotective benefits, protecting the heart, brain, blood vessels, and other organs from damage (Hsu et al., 2017; Ren, 2022; Si et al., 2022; Wang, 2023; Hou et al., 2024). Thus, RCE may provide clinical benefits to patients in the treatment of symptoms of altitude sickness such as headache, nausea, anorexia, gastrointestinal discomfort, insomnia, fatigue, and hair loss (Zhang and Shu, 2011; Liu et al., 2022).

Currently, numerous clinical studies on RCE for AHAD have been published. Nevertheless, only a single systematic review of the quality of its research findings and methodologies exists, and it was published at an early stage (Cao et al., 2015). This suggested that the translation of evidence concerning the efficacy of RCE in treating AHAD has not been adequately addressed (Liu and Gong, 2024; Zhang et al., 2024). Consequently, this study conducted a systematic review and meta-analysis to assess the efficacy and safety of RCE in the treatment of AHAD, with the objective of providing robust and practical evidence for clinical application.

## 2 Materials and methods

### 2.1 Study registration and reporting guideline

The protocol for this systematic review, registered with PROSPERO (CRD42024593081) on 1 October 2024, adheres to the PRISMA 2020 guidelines (Page et al., 2021).

### 2.2 Ethical statement

Since this study is a literature review, it did not require ethical approval.

### 2.3 Inclusion criteria

#### 2.3.1 Type of study

The study included randomized controlled trials (RCTs) that were published in either English or Chinese.

#### 2.3.2 Participants

The diagnosis of followed the diagnostic criteria outlined in the *2018 Lake Louise Acute Mountain Sickness Score* (Roach et al., 2018) and *Guidelines for the Diagnosis, Prevention and Treatment of High Altitude Disease* (2014) (Wu, 2014). No restrictions exist regarding age, gender, nationality, birth location, or ethnic origin. The nomenclature, classification and relevant diagnostic criteria are listed in Supplementary Material S1.

#### 2.3.3 Intervention and comparison

The control group was given either conventional western medicine (WM) or placebo. Western medicine encompasses conventional drugs, excluding traditional Chinese medicine (TCM), such as acetazolamide, dexamethasone, and aminophylline, used for AHAD. The intervention group was administered RCE (all dosage forms, such as capsules and aqueous extracts, were included) or a combination of WM and RCE, without consideration for the dosage, duration, or frequency of RCE administration. Comprehensive information regarding the RCE is available in Supplementary Material S2.

#### 2.3.4 Outcomes

1) Primary outcomes: arterial oxygen saturation (SaO_2_) and arterial partial pressure of oxygen (PaO_2_).
2) Secondary outcomes: total clinical efficacy, systolic blood pressure (SBP), diastolic blood pressure (DBP) and heart rate (HR).

The criteria for evaluating total clinical efficacy are as follows: ①Significant Effective: the patient’s symptoms and signs completely recovered; ②Effective: the patient’s symptoms and signs were basically recovered; ③Invalid: the patient’s symptoms and signs did not return to normal, and his condition worsened. Total clinical efficacy = number of effective cases/total number of cases × 100%.

3) Safety outcome: The incidence of adverse events.
4) Other outcomes: To comprehensively assess the efficacy and safety of RCE in treating AHAD, all reported outcomes from the RCTs were included in the analysis.

### 2.4 Exclusion criteria

1) Incomplete or inaccurate data, including missing baseline data, mean, SD, etc.
2) Interventions included other TCM components or therapies other than RCE.
3) Single-arm studies.
4) For duplicate publications, only one was included.

### 2.5 Information source and search strategy

Eight databases, including CNKI, VIP, WF, SinoMed, PubMed, Embase, Cochrane Library, and Web of Science, were comprehensively searched for RCTs from their inception until September 2024. Search conducted between September 1 and 10, 2024. The reference lists from the included trials were manually reviewed to find any additional relevant studies. The search strategy is detailed using PubMed as an example. Strategies for other databases can be found in Supplementary Material S3.

#1″Altitude Sickness” [MeSH Terms] OR “Altitude Diseases” [Title/Abstract] OR “Sickness, Altitude” [Title/Abstract] OR “Diseases, Altitude” [Title/Abstract] OR “Altitude Hypoxia” [Title/Abstract] OR “Altitude Hypoxias” [Title/Abstract] OR “Hypoxia, Altitude” [Title/Abstract] OR “Mountain Sickness” [Title/Abstract] OR “Sickness, Mountain” [Title/Abstract].

#2″*R. crenulata*” [MeSH Terms] OR “*Rhodiola rosea*” [Title/Abstract] OR “*Roseroot*” [Title/Abstract] OR “*Roseroots*” [Title/Abstract] OR “Hongjingtian” [Title/Abstract] OR “Hong jing tian” [Title/Abstract].

#3″randomized controlled trial” [Publication type] OR “randomized clinical trial” [Publication type] OR “randomized trial” [Publication type] OR “clinical trial” [Publication type] OR “randomized controlled trial” [Title/Abstract] OR “randomized clinical trial” [Title/Abstract] OR “randomized trial” [Title/Abstract] OR “clinical trial” [Title/Abstract].

#4 #1 AND #2 AND #3.

### 2.6 Study screening and data extraction

The study’s screening protocol involved: 1) Reviewing titles and abstracts to choose studies that met the inclusion criteria; 2) Examining the full text if additional information was required. Design the standard data extraction table, including: title, primary author, publication year, source, sample size, age, gender, disease diagnosis, diagnostic criteria, disease duration, course of treatment, follow-up and outcomes. If complete data were unavailable, the authors would be emailed; lack of response would result in exclusion from the study. The processes of study screening, data extraction, and risk of bias assessment were independently conducted by two researchers. Discrepancies were resolved through discussion or, if necessary, by consulting a third researcher.

### 2.7 Risk of bias assessment

The risk of bias of RCTs was assessed by using the Cochrane Collaboration Risk of Bias tool (https://methods.cochrane.org/bias/risk-bias-tool) for randomized controlled trials 2.0 (RoB 2.0) (Sterne et al., 2019). Bias was assessed in each of the five domains using specific signaling questions:

1) randomization process (selection bias);
2) deviations from the intended interventions (performance bias);
3) missing outcome data (attrition bias);
4) measurement of the outcome (detection bias);
5) selection of the reported outcome (reporting bias).

Bias risk in each domain was described as “low risk,” “some concerns,” or “high risk.” The overall risk of bias for each study was determined by assessing the risk of bias in each domain.

### 2.8 Statistical analysis

The meta-analysis was conducted using Review Manager software (Cochrane Collaboration, version 5.4) along with Stata 15.0. For dichotomous variables, the relative risk (RR) was used as the effect measure for analysis. For continuous variables, meta-analysis used the mean and standard deviation (SD) of pre- and post-treatment differences (Mean ± SD), employing the mean difference (MD) or standardized mean difference (SMD) as effect statistics. When the outcome was evaluated utilizing consistent measurement methods and units, MD was employed; otherwise, SMD was chosen. Statistical analysis results were expressed using 95% confidence intervals (CI). Statistical heterogeneity was evaluated using the *P* value and the *I* ^*2*^ statistic. A threshold of *I* ^*2*^ ≤ 50% and *P* > 0.05 indicated low heterogeneity among the included studies, thereby warranting the use of a fixed-effects model for the synthesis of results. Otherwise, it was considered that the included studies had high heterogeneity, and subgroup or sensitivity analyses would be employed to investigate possible origins of this heterogeneity. If the source of heterogeneity cannot be determined, the outcomes will be combined using a random-effects model. In this study, the I^2^ statistic for all primary and secondary outcomes exceeded 50%. Subsequent sensitivity analyses, which accounted for variables such as age, dosage form, intervention dosage, and studies identified as having a high risk of bias, did not result in a significant reduction in heterogeneity. Consequently, a random-effects model was utilized for the meta-analysis.

For analyses incorporating data from over 10 studies, funnel plots and Egger’s test were employed to evaluate the presence of publication bias. A P-value of less than 0.05 from Egger’s test was interpreted as evidence suggesting the existence of publication bias. Due to the fact that none of the analyzed outcomes encompassed more than ten studies, an assessment of publication bias was not performed. This study aims to investigate the efficacy of three distinct interventions: RCE vs. placebo, RCE vs. WM and RCE + WM vs. WM. In addition, subgroup analyses will be performed to assess the efficacy of varying treatment durations (≤7 days and >7 days) utilizing the same intervention.

### 2.9 Certainty of evidence

The certainty of the evidence was evaluated using the Grading of Recommendations Assessment, Development, and Evaluation (GRADE) approach, which takes into account five factors: risk of bias, imprecision, inconsistency, indirectness, and publication bias. The principal findings were delineated in the Summary of Findings table, which was generated using the GRADE Pro GDT software (http://gradepro.org). Evidence certainty is ranked as high, moderate, low, or very low based on the available evidence (Balshem et al., 2011).

## 3 Results

### 3.1 Study screening

**FIGURE 1:**
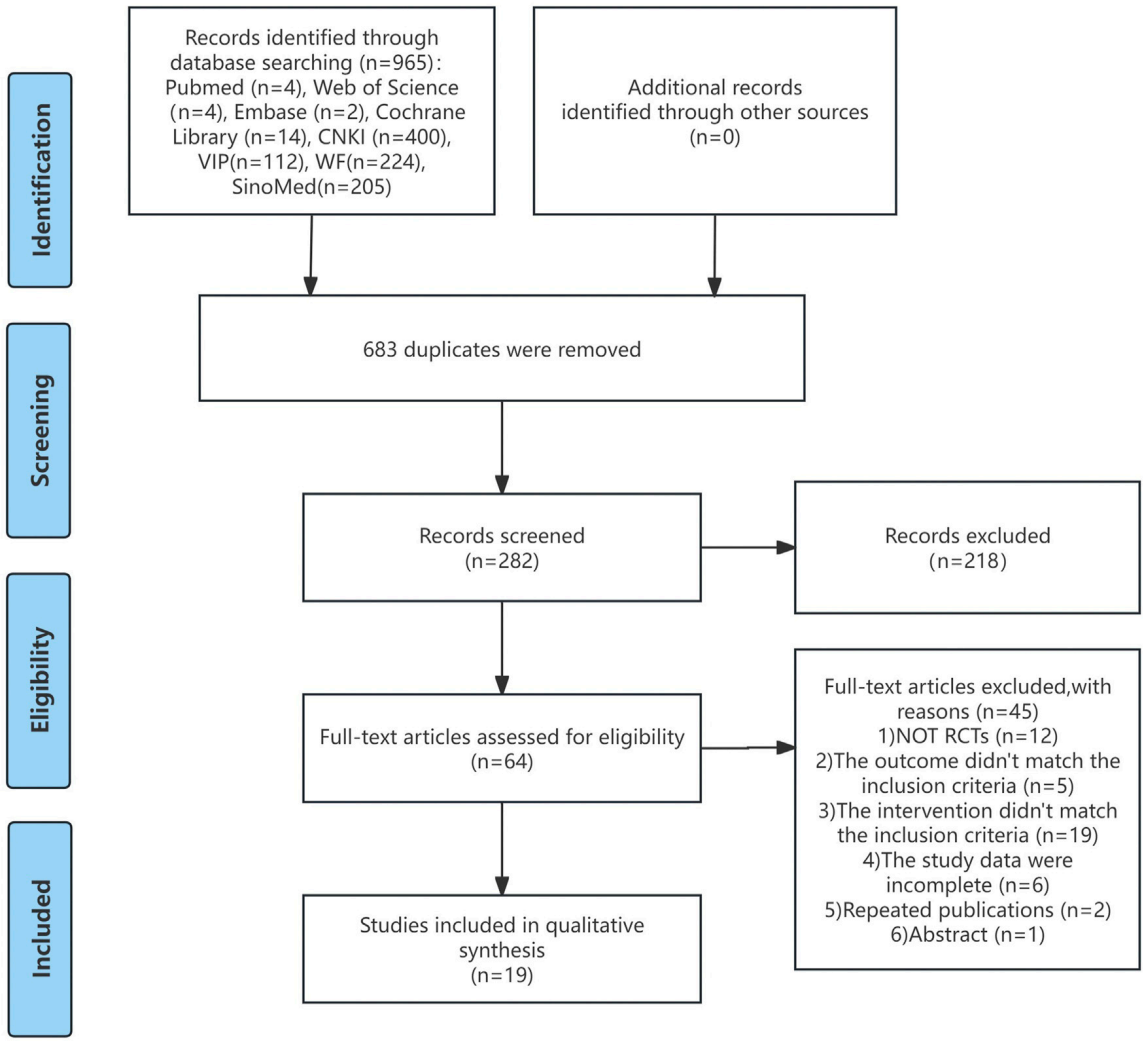
Flow diagram of study screening.

A comprehensive search across eight databases yielded 965 articles. Following the removal of 683 duplicate entries, title and abstract screening resulted in the exclusion of 218 studies. A full-text assessment led to the exclusion of 45 articles that failed to meet the inclusion criteria, including non-RCTs, misaligned interventions and outcomes, incomplete data, duplicates, and reviews. Ultimately, 19 RCTs, all published in Chinese, were included in the analysis. Figure 1 depicts the study screening process.

### 3.2 Study characteristics

Table 1 presents a comprehensive summary of the characteristics of the studies included in this analysis. All 19 RCTs published between 2003 and 2020. All studies are from China, which limits global representativeness. These RCTs collectively involved a total of 1,690 participants, with 871 individuals assigned to the intervention group and 819 to the control group. Four RCTs with 314 participants compared RCE with WM, thirteen RCTs with 1,244 participants compared RCE with placebo (one study compared RCE with both WM and placebo), and three RCTs with 141 participants compared RCE plus WM with WM. WM included acetoamide, aminophylline, edaravone, trimetazidine, among other conventional pharmaceuticals. Placebo included medical starch capsules and purified water. The duration of the treatment regimen varied from 5 days to 3 months. No significant disparities were observed in the baseline characteristics across all the studies.

**TABLE 1:** Characteristics of included studies.

Included studies	Sample size (T/C)	Age (T/C)	Intervention		Treatment duration (days)	Treatment dosage	Outcome
			T	C			
Niu et al. (2003)	29/30/30	18.4	RCE/RCE	placebo	6	2 particles per time, tid	⑧⑪㉔
Wang et al. (2003)	29/30/30	-	RCE/RCE	placebo	6	2 particles per time, tid	㊹
Cai et al. (2003)	37/37	18–22	RCE	placebo	7	0.4g per pill, 2 pills per time, bid	①②③⑥⑦⑩⑮⑱㉔㉜㉞㉟
Niu et al. (2006)	50/50	17–21	RCE	placebo	7	0.2g per pill, 3 pills per time, bid	㉜
Yu et al. (2006)	50/50	17–21	RCE	placebo	8	0.2g per pill, 3 pills per time, bid	④⑤㉙㉚
Yang et al. (2008)	20/20	58.5 ± 8.0	RCE + WM	WM	21	2g per time, 3 times per day	②⑨㊷
Li et al. (2008)	9/13/19	26.0 ± 4.5/28.0 ± 3.7/24.0 ± 5.2	RCE	WM/placebo	-	0.25–0.5g per times, qd	①⑥
Hao et al. (2008)	10/10	21.6 ± 2.0	RCE	placebo	14	10 mL per time, bid	㊲㊳㊴㊵㊶
He (2011)	40/40	37.7 ± 6.2/37.3 ± 6.5	RCE	WM	7	50 mL per time, tid	③⑦
Yu et al. (2012)	110/110	18.7 ± 2.4	RCE	placebo	14	2 particles per time, tid	①
Chiu et al. (2013)	48/54	35.8 ± 10.0/36.3 ± 10.4	RCE	placebo	9	0.4g per pill, 2 pills per time, qd	⑦⑯
Hu et al. (2014)	25/23	51–81	RCE + WM	WM	15	2 particles per time, tid	㊱㊸
Wei et al. (2014)	26/27	13–72	RCE + WM	WM	15	2 particles per time, tid	③㊱
Bi et al. (2015)	35/39	-	RCE	placebo	40	2 particles per time, bid	⑰⑲
Duan et al. (2015)	24/24	18.3 ± 0.4/18.1 ± 0.3	RCE	placebo	5	0.38g per pill, 2 pills per time, bid	④⑤⑥⑨⑫㉚⑯㊹
Lei et al. (2015)	50/50	35 ± 1.8/36 ± 2.4	RCE	placebo	10	0.6g per pill, 4 pills per time, tid	⑥⑩⑯㉕㉛㉜㊹
Tian et al. (2015)	60/60	18.41 ± 1.41/18.37 ± 1.71	RCE	WM	7	2 pills per time, bid	①②④⑭⑮⑰⑤⑧⑪⑬⑱⑳㉑㉒㉓㉖㉗㉘㉝㊺
Li et al. (2017)	100/100	26.90 ± 6.27	RCE	placebo	6	0.38g per pill, 2 pills per time, tid	④⑤
Zhou (2020)	46/46	43.5 ± 2.6/42.5 ± 2.4	RCE	WM	7	0.5g per pill, 2 pills per time, bid	③

Abbreviations: T, treatment group; C, control group; RCE, *rhodiola crenulata extract*; WM, western medicine; qd, one time per day; bid, 2 times per day; tid, 3 times per day. ①, arterial oxygen saturation (SaO_2_); ②, arterial partial pressure of oxygen (PaO_2_); ③, total clinical efficacy; ④, systolic blood pressure (SBP); ⑤, diastolic blood pressure (DBP); ⑥, heart rate (HR); ⑦, adverse events; ⑧, breath-holding index (BHI); ⑨, mean pulmonary arterial pressure (MPAP); ⑩, breathing rate (RR); ⑪, ventilation efficiency index (VEI); ⑫, pulmonary arterial systolic pressure (PASP); ⑬, vital capacity (VC); ⑭, alveolar-arterial oxygen partial pressure difference (A-aDO_2_); ⑮, arterial partial pressure of carbon dioxide (PaCO_2_); ⑯, pulse oxygen saturation (SpO_2_); ⑰, blood lactate (BLA); ⑱, potential of hydrogen (pH); ⑲, buffuer excess (BE); ⑳, carbonic acid hydrogen radical (HCO_3_-); ㉑, malondialdehyde (MDA); ㉒, nitric oxide (NO); ㉓, superoxide dismutase (SOD); ㉔, pulse rate (PR); ㉕, cardiac index (CI); ㉖, cardiac function index (CFI); ㉗, catalase (CAT); ㉘, creatine kinase (CK); ㉙, pulse pressure difference (PP); ㉚, mean arterial pressure (MAP); ㉛, systemic vascular resistance (SVR); ㉜, acute high altitude disease score (AHAD, sore); ㉝, high altitude adaptation index (HAAI); ㉞, headache score; ㉟, vomiting score; ㊱, volume change of cerebral oedema; ㊲, main wave amplitude of cerebral blood flow; ㊳, time of cerebral blood flow up; ㊴, time of cerebral blood flow inflow; ㊵, cereblood flow into volume velocity; ㊶, resistance index of cerebral blood flow inflow; ㊷, basic fibroblast growth factor (bFGF); ㊸, haemoglobin (Hb); ㊹, incidence of acute high altitude disease (AHAD, incidence); ㊹, occurrence of acute hypoxia symptoms; ㊺, hydrogen peroxide (H_2_O_2_).

### 3.3 Risk of bias assessment

**FIGURE 2:**
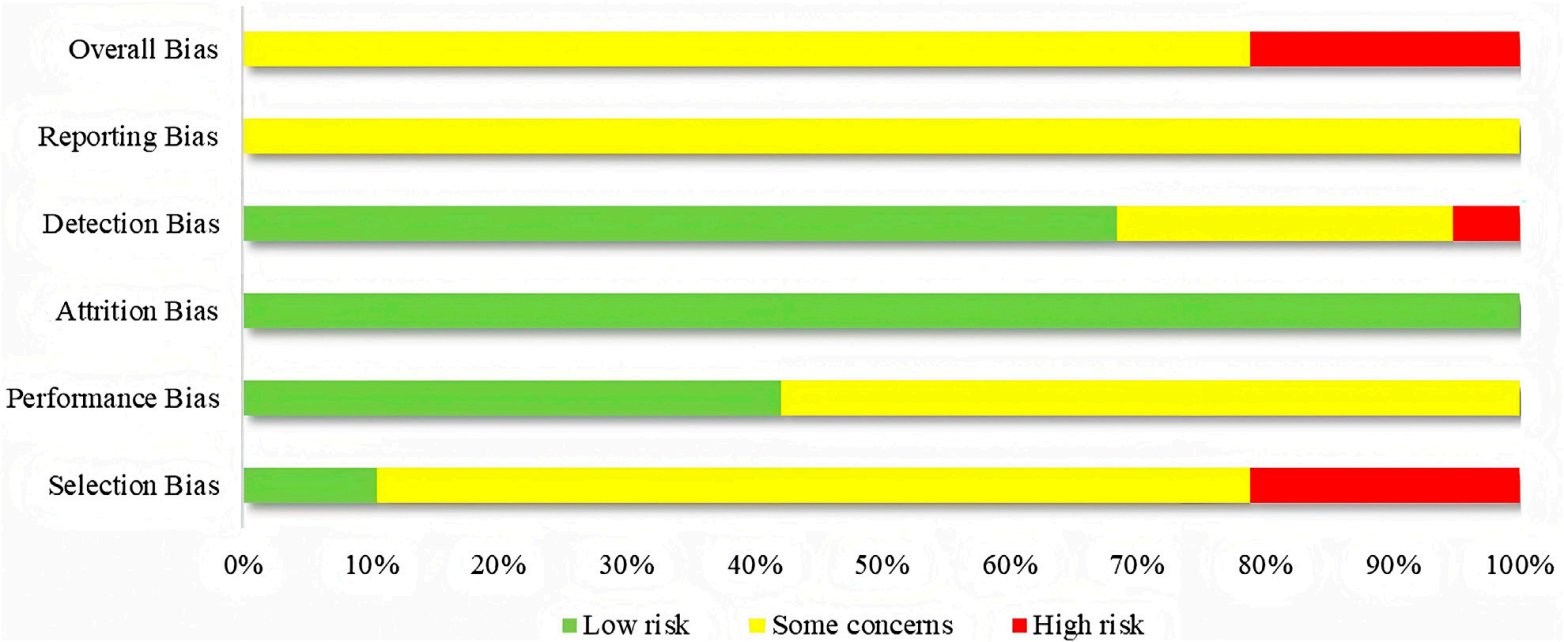
Risk of bias assessment for included studies.

The 19 studies were assessed based on the five domains of ROB 2.0. Seventeen studies exhibited issues with the randomization process, with 13 studies only mentioning randomization without detailing the method of implementation. Furthermore, none of the 17 studies looked into how allocation concealment was implemented. Eleven studies faced issues with deviations from the intended interventions, potentially resulting in a divergence from the intended intervention due to the lack of blinding. In terms of missing outcome data, none of the 19 studies reported any dropout cases, and all outcome data were complete. Issues with measurement of the outcome were present in six studies, with one study being at high risk of bias due to vague criteria for measuring outcomes. All 19 studies faced issues with selection of the reported results, as none of the studies had established a predetermined protocol. Among the overall risk of bias, 15 studies were rated as some concerns, and four studies were rated as high risk. The findings suggested that the principal limitations affecting the quality of the study were attributable to biases in selection and reporting. Furthermore, almost all studies were published in Chinese, potentially introducing language bias and limiting the generalizability of the findings. Figure 2 presents the assessment of bias risk for the included studies.

### 3.4 Meta-analysis results

#### 3.4.1 Primary outcomes

##### 3.4.1.1 SaO_2_

**FIGURE 3:**
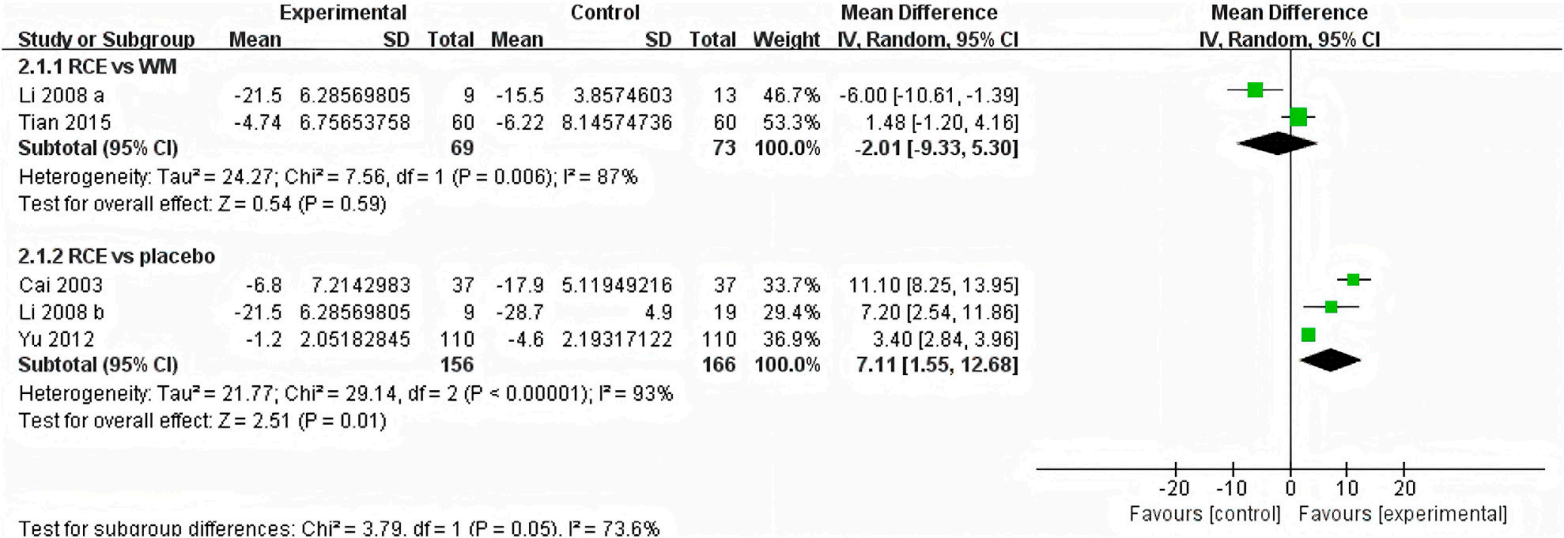
Forest plot of the effect of *Rhodiola crenulata* extract (RCE) vs. western medicine (WM) and RCE vs. placebo on arterial oxygen saturation (SaO_2_).

SaO_2_ was reported in 4 RCTs including 464 participants (Cai et al., 2003; Li et al., 2008; Yu et al., 2012; Tian et al., 2015), with three being two-arm trials and one a three-arm trial (Figure 3). Two RCTs reported RCE vs. WM (including 142 participants). The heterogeneity test indicated significant heterogeneity among the studies (P = 0.006, *I* ^*2*^ = 87%). The sensitivity analysis showed that removing any single study did not decrease the significant heterogeneity of the combined results. Therefore, a random-effects model was employed to pool the results. The results of the meta-analysis revealed that there was no statistically significant difference between the two groups [MD = −2.01, 95%CI (−9.33, 5.30), *p* = 0.59], implying that RCE showed efficacy comparable to WM in improving SaO_2_ levels. Three RCTs reported RCE vs. placebo (including 322 participants). The heterogeneity test indicated significant heterogeneity among the studies (P < 0.00001, *I* ^*2*^ = 93%). The sensitivity analysis showed that removing any single study did not decrease the significant heterogeneity of the combined results. Therefore, a random-effects model was employed to pool the results. The results of the meta-analysis revealed that RCE demonstrated significant efficacy in improving SaO_2_ [MD = 7.11, 95%CI (1.55, 12.68), *p* = 0.01]. Despite the statistical improvement in SaO_2_ with RCE compared to placebo, the high heterogeneity and risk of bias among the studies prevent a robust conclusion.

##### 3.4.1.2 PaO_2_

**FIGURE 4:**
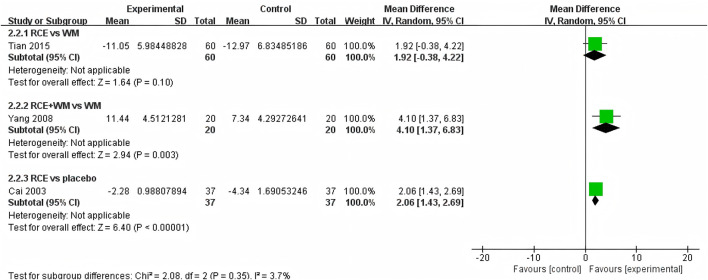
Forest plot of the effect of *Rhodiola crenulata* extract (RCE) vs. western medicine (WM), RCE combined with WM vs. WM and RCE vs. placebo on arterial partial pressure of oxygen (PaO_2_).

PaO_2_ was reported in 3 RCTs including 234 participants (Cai et al., 2003; Yang et al., 2008; Tian et al., 2015) (Figure 4). One RCT reported RCE vs. WM (including 120 participants), the meta-analysis indicated that there was no significant difference between the two groups [MD = 1.92, 95%CI (−0.38, 4.22), *p* = 0.10], implying that RCE showed efficacy comparable to WM in improving PaO2 levels. One RCT reported RCE + WM vs. WM (including 40 participants), the meta-analysis indicated that RCE demonstrated significant efficacy in improving PaO_2_ levels [MD = 4.10, 95%CI (1.37. 6.83), *p* = 0.003]. One RCT reported RCE vs. placebo (including 74 participants), the meta-analysis indicated that RCE demonstrated significant efficacy in improving PaO_2_ levels [MD = 2.06, 95%CI (1.43, 2.69), *p* < 0.00001]. Despite the statistical improvement in PaO_2_ with RCE combined with WM compared to WM, as well as between RCE compared to placebo, the high heterogeneity and risk of bias among the studies prevent a robust conclusion.

#### 3.4.2 Secondary outcomes

##### 3.4.2.1 Total clinical efficacy

**FIGURE 5:**
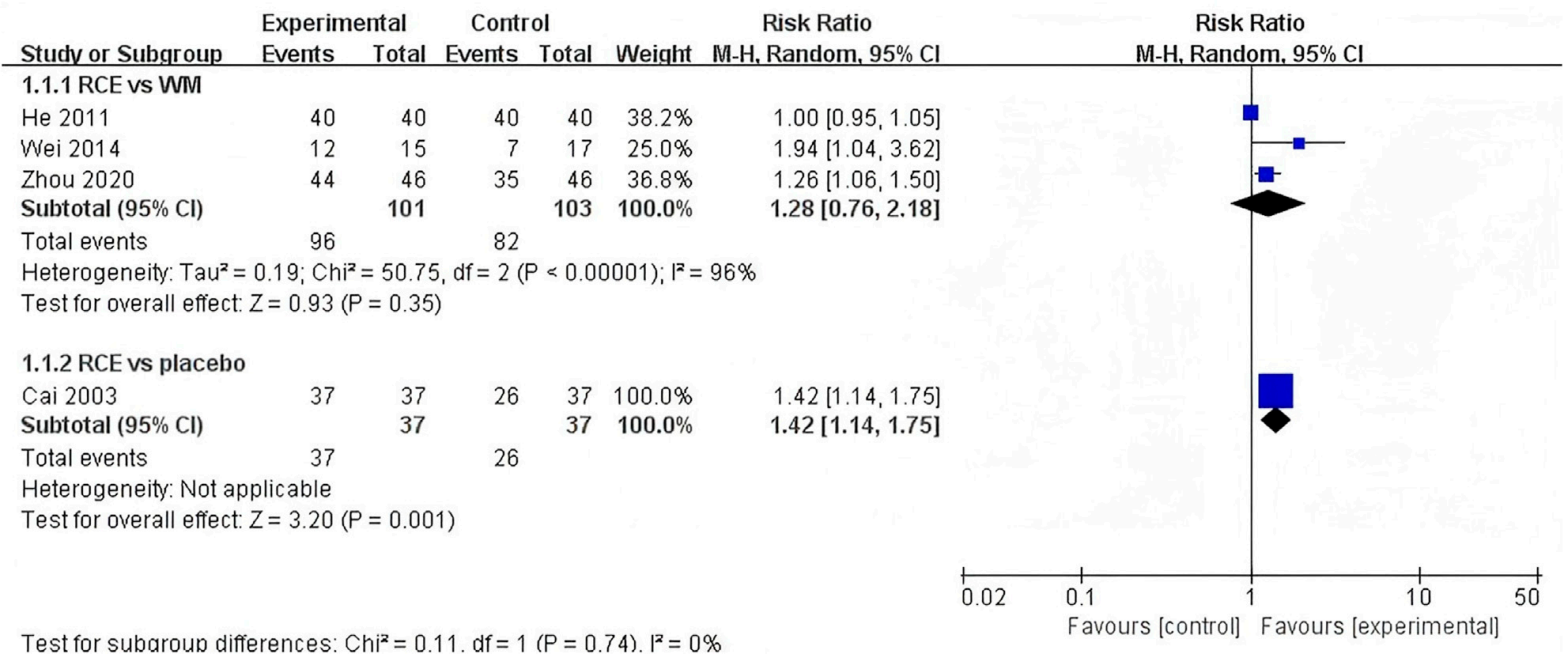
Forest plot of the effect of *Rhodiola crenulata* extract (RCE) vs. western medicine (WM) and RCE vs. placebo on total clinical efficacy.

The total clinical efficacy was reported in 4 RCTs including 278 participants (Cai et al., 2003; He, 2011; Wei et al., 2014; Zhou, 2020) (Figure 5). Three RCTs reported RCE vs. WM (including 204 participants). The heterogeneity test indicated significant heterogeneity among the studies (P < 0.00001, *I* ^*2*^ = 96%). The sensitivity analysis showed that removing any single study did not decrease the significant heterogeneity of the combined results. Therefore, a random-effects model was employed to pool the results. The results of the meta-analysis revealed that there was no statistically significant difference between the two groups [RR = 1.28, 95%CI (0.76, 2.18), *p* = 0.35], suggesting that RCE showed efficacy comparable to WM on total clinical efficacy. One RCT reported RCE vs. placebo (including 74 participants), the meta-analysis revealed a significant improving effect of RCE on total clinical efficacy [RR = 1.42, 95%CI (1.14, 1.75), *p* = 0.001]. Despite the statistical improvement on total clinical efficacy compared to placebo, the small sample size of the study and risk of bias among the studies prevent a robust conclusion.

##### 3.4.2.2 SBP

**FIGURE 6:**
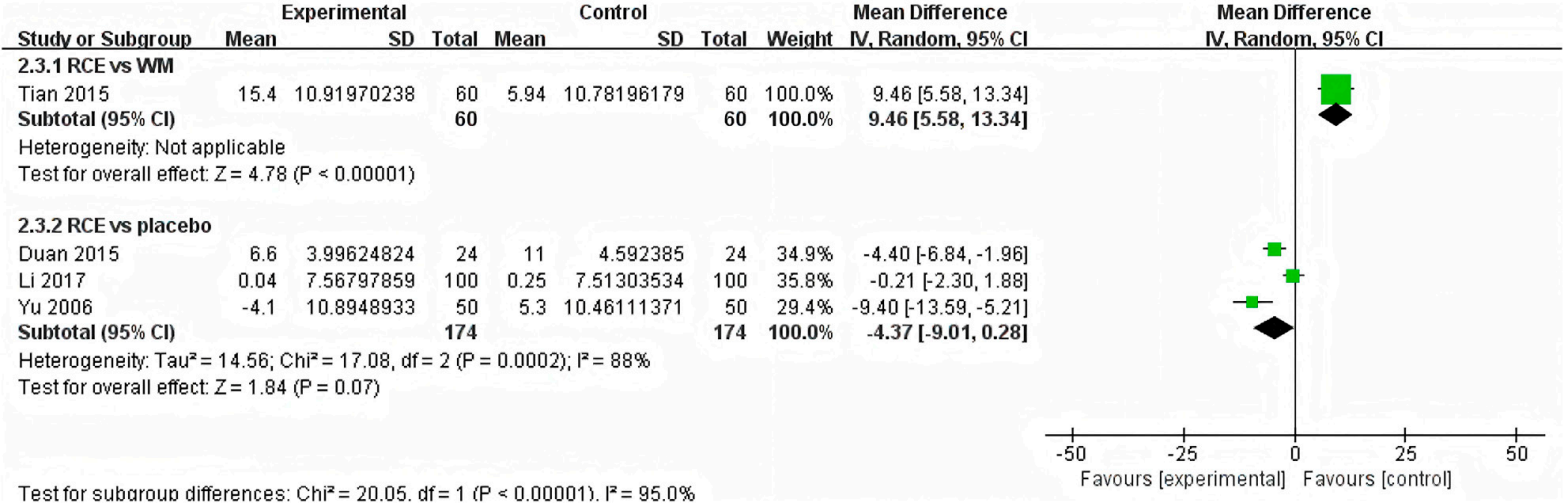
Forest plot of the effect of *Rhodiola crenulata* extract (RCE) vs. western medicine (WM) and RCE vs. placebo on systolic blood pressure (SBP).

SBP was reported in 4 RCTs including 468 participants (Yu et al., 2006; Duan et al., 2015; Tian et al., 2015; Li et al., 2017) (Figure 6). One RCT reported RCE vs. WM (including 120 participants), the meta-analysis indicated that WM exhibits superior efficacy compared to RCE in reducing SBP [MD = 9.46, 95%CI (5.58, 13.34), *p* < 0.00001]. Three RCTs reported RCE vs. placebo (including 348 participants). The heterogeneity test indicated significant heterogeneity among the studies (P = 0.0002, *I* ^*2*^ = 88%). The sensitivity analysis showed that removing any single study did not decrease the significant heterogeneity of the combined results. Therefore, a random-effects model was employed to pool the results. The results of the meta-analysis revealed that there was no statistically significant difference between the two groups [MD = −4.37, 95%CI (−9.01, 0.28), *p* = 0.07]. Nevertheless, it is important to acknowledge that the substantial heterogeneity and potential for bias present in the studies may hinder the formulation of robust conclusions.

##### 3.4.2.3 DBP

**FIGURE 7:**
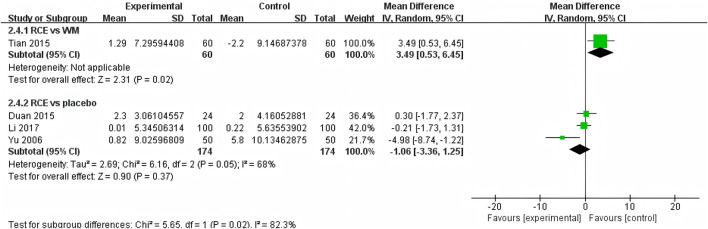
Forest plot of the effect of *Rhodiola crenulata* extract (RCE) vs. western medicine (WM) and RCE vs. placebo on diastolic blood pressure (DBP).

DBP was reported in 4 RCTs including 468 participants (Yu et al., 2006; Duan et al., 2015; Tian et al., 2015; Li et al., 2017) (Figure 7). One RCT reported RCE vs. WM (including 120 participants), the meta-analysis indicated that WM exhibits superior efficacy compared to RCE in reducing DBP [MD = 3.49, 95%CI (0.53, 6.45), *p* = 0.02]. Three RCTs reported RCE vs. placebo (including 348 participants). The heterogeneity test indicated significant heterogeneity among the studies (P = 0.05, *I* ^*2*^ = 68%). The sensitivity analysis showed that removing any single study did not decrease the significant heterogeneity of the combined results. Therefore, a random-effects model was employed to pool the results. The results of the meta-analysis revealed that there was no statistically significant difference between the two groups [MD = −1.06, 95%CI (−3.36, 1.25, *p* = 0.37]. Nevertheless, it is important to acknowledge that the substantial heterogeneity and potential for bias present in the studies may hinder the formulation of robust conclusions.

##### 3.4.2.4 HR

**FIGURE 8:**
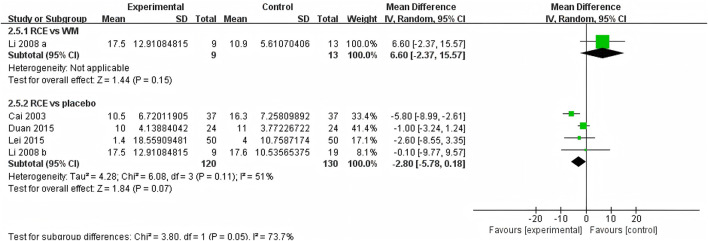
Forest plot of the effect of *Rhodiola crenulata* extract (RCE) vs. western medicine (WM) and RCE vs. placebo on heart rate (HR).

HR was reported in 4 RCTs including 272 participants (Cai et al., 2003; Li et al., 2008; Duan et al., 2015; Lei et al., 2015), with three being two-arm trials and one a three-arm trial (Figure 8). One RCT reported RCE vs. WM (including 22 participants), the meta-analysis indicated that there was no significant difference between the two groups [MD = 6.60, 95%CI (−2.37, 15.57), *p* = 0.15]. Three RCTs reported RCE vs. placebo (including 250 participants). The heterogeneity test indicated significant heterogeneity among the studies (P = 0.11, *I* ^*2*^ = 51%). The sensitivity analysis showed that removing any single study did not decrease the significant heterogeneity of the combined results. Therefore, a random-effects model was employed to pool the results. The results of the meta-analysis indicated that there was no statistically significant difference observed between the two groups [MD = −2.80, 95%CI (−5.78, 0.18, *p* = 0.07]. Nevertheless, it is important to acknowledge that the substantial heterogeneity and potential for bias present in the studies may hinder the formulation of robust conclusions.

A summary table of primary and secondary outcomes are shown in Table 2.

**TABLE 2:** Meta-analysis results of primary and secondary outcomes.

Outcome	Subgroup	Number of study	Sample size (T/C)	Measures	Effect estimate (95 %CI)	Heterogeneity (*I* ^*2*^)	*P*_interaction_
SaO_2_	RCE vs. WM	2	69/73	MD (Random)	−2.01 [-9.33, 5.30]	87%	0.59
	RCE vs. placebo	3	156/166	MD (Random)	7.11 [1.55, 12.68]	93%	0.01
PaO_2_	RCE vs. WM	1	60/60	MD (N/A)	1.92 [-0.38, 4.22]	-	0.10
	RCE + WM vs. WM	1	20/20	MD (N/A)	4.01 [1.37, 6.83]	-	0.003
	RCE vs. placebo	1	37/37	MD (N/A)	2.06 [1.43, 2.69]	-	<0.00001
Total clinical efficacy	RCE vs. WM	3	101/103	RR (Random)	1.28 [0.76, 2.18]	96%	0.35
	101 RCE vs. placebo	1	119/118	RR (N/A)	1.42 [1.14, 1.75]	-	0.001
SBP	RCE vs. WM	1	60/60	MD (N/A)	9.40 [5.58, 13.34]	-	<0.00001
	RCE vs. placebo	3	174/174	MD (Random)	−4.37 [-9.01, 0.28]	88%	0.07
DBP	RCE vs. WM	1	60/60	MD (N/A)	3.49 [0.53, 6.45]	-	0.02
	RCE vs. placebo	3	174/174	MD (Random)	−1.06 [-3.36, 1.25]	68%	0.37
HR	RCE vs. WM	1	9/13	MD (N/A)	6.60 [-2.37, 15.57]	-	0.15
	RCE vs. placebo	4	120/130	MD (Random)	−2.80 [-5.78, 0.18]	51%	0.07

Abbreviations: MD, mean difference; RR, relative risk; CI, confidence interval; *P*_interaction_, *P* for interaction; T, treatment group; C, control group; SaO_2_, arterial oxygen saturation; PaO_2_, arterial partial pressure of oxygen; SBP, systolic blood pressure; DBP, diastolic blood pressure; HR, heart rate.

#### 3.4.3 Other outcomes

The meta-analysis results indicated that RCE demonstrated efficacy across these outcomes, including pulmonary function (increasing VEI, reducing MPAP and PASP), cardiovascular function (reducing PP, decreasing CK and increasing CFI), blood gas analysis (decreasing PaCO_2_), biochemical indices (increasing NO, decreasing H_2_O_2_ and Hb) and evaluation index of altitude disease (increasing HAAI, decreasing vomiting score and the incidence of AHAD). However, the efficacy of reducing CAT and MDA levels was limited. The remaining results did not demonstrate a statistically significant difference. The results are shown in Table 3.

**TABLE 3:** Meta-analysis results of other outcomes.

Classification	Outcome	Number of study	Sample size (T/C)	Measures	Effect estimate (95 %CI)	Heterogeneity (*I* ^*2*^)	*P*_interaction_
Pulmonary function	BHI	2 (3 Groups of data)	119/120	MD (Random)	0.03 [-0.09, 0.15]	62%	0.65
	VEI	2 (3 Groups of data)	119/120	MD (Random)	0.16 [0.02, 0.31]	59%	0.03
	Breathing rate	2	87/87	MD (Random)	−1.92 [-5.44, 1.60]	90%	0.29
	MPAP	2	44/44	MD (Fixed)	−1.92 [-2.96, −0.88]	0%	0.0003
	PASP	1	24/24	MD (N/A)	−2.50 [-3.61, −1.39]	-	<0.00001
	VC	1	60/60	MD (N/A)	250.66 [-152.57, 653.89]	-	0.22
	A-aDO_2_	1	60/60	MD (N/A)	−0.63 [-1.79, 0.53]	-	0.29
Cardiovascular function	PR	2 (3 Groups of data)	96/90	MD (Random)	−0.69 [-6.64, 5.26]	67%	0.82
	MAP	2	74/74	MD (Random)	−1.61 [-9.55, 6.32]	89%	0.69
	Cardiac index	1	50/50	MD (N/A)	0.02 [-0.11, 0.15]	-	0.76
	CFI	1	60/60	MD (N/A)	2.19 [0.77, 3.61]	-	0.002
	CK	1	60/60	MD (N/A)	−6.03 [-11.10, −0.96]	-	0.02
	PP	1	50/50	MD (N/A)	−6.33 [-10.58, −2.08]	-	0.004
	SVR	1	50/50	MD (N/A)	6.56 [-9.36, 22.48]	-	0.42
Cerebral function	Volume change of cerebral oedema	2	43/42	MD (Random)	−2.37 [-6.36, 1.62]	85%	0.24
	Main wave amplitude of cerebral blood flow	1	10/10	MD (N/A)	−0.02 [-0.07, 0.02]	-	0.27
	Time of cerebral blood flow up	1	10/10	MD (N/A)	0.01 [-0.03, 0.05]	-	0.61
	Time of cerebral blood flow inflow	1	10/10	MD (N/A)	−0.00 [-0.03, 0.03]	-	0.95
	Cereblood flow into volume velocity	1	10/10	MD (N/A)	0.19 [-0.17, 0.55]	-	0.31
	Resistance index of cerebral blood flow inflow	1	10/10	MD (N/A)	0.01 [-0.10, 0.11]	-	0.87
Blood gas analysis	SpO_2_	3	122/128	MD (Random)	0.20 [-1.31, 1.71]	74%	0.79
	PaCO_2_	2	97/97	MD (Fixed)	−0.27 [-0.48, −0.06]	0%	0.01
	BLA	2	95/99	MD (Random)	2.60 [-2.56, 7.75]	100%	0.32
	PH	2	97/97	MD (Random)	0.01 [-0.01, 0.03]	89%	0.42
	BE	1	35/39	MD (N/A)	0.05 [-1.13, 1.23]	-	0.93
	HCO_3_-	1	60/60	MD (N/A)	−0.60 [-1.22, 0.02]	-	0.06
Biochemical indices	bFGF	1	20/20	MD (N/A)	−6.00 [-12.80, 0.80]	-	0.08
	Hb	1	25/25	MD (N/A)	−13.38 [-15.93, −10.83]	-	<0.00001
	CAT	1	60/60	MD (N/A)	1.11 [0.76, 1.46]	-	<0.00001
	H_2_O_2_	1	60/60	MD (N/A)	−15.38 [-18.04, −12.72]	-	<0.00001
	NO	1	60/60	MD (N/A)	204.77 [198.76, 210.78]	-	<0.00001
	MDA	1	60/60	MD (N/A)	0.68 [0.46, 0.90]	-	<0.00001
	SOD	1	60/60	MD (N/A)	0.52 [-0.11, 1.15]	-	0.01
Evaluation index of altitude disease	AHAD sore	3	137/137	MD (Random)	−0.20 [-2.63, 2.22]	96%	0.87
	HAAI	1	60/60	MD (N/A)	0.06 [0.01, 0.11]	-	0.02
	Headache score	1	37/37	MD (N/A)	−0.55 [-1.38, 0.28]	-	0.19
	Vomiting score	1	37/37	MD (N/A)	−0.83 [-1.51, −0.15]	-	0.02
	AHAD incidence	2	74/74	RR (Fixed)	0.59 [0.43, 0.82]	0%	0.002
	Occurrence of acute hypoxia symptoms	1 (2 Groups of data)	59/60	RR (Fixed)	0.71 [0.44, 1.14]	0%	0.16

Abbreviations: MD, mean difference; RR, relative risk; CI, confidence interval; *P*_interaction_, *P* for interaction; T, treatment group; C, control group; BHI, breath-holding index; MPAP, mean pulmonary arterial pressure; VEI, ventilation efficiency index; PASP, pulmonary arterial systolic pressure; VC, vital capacity; A-aDO_2_, alveolar-arterial oxygen partial pressure difference; PaCO_2_, arterial partial pressure of carbon dioxide; SpO_2_, pulse oxygen saturation; BLA, blood lactate; pH, potential of hydrogen; BE, buffuer excess; HCO_3_-, carbonic acid hydrogen radical; MDA, malondialdehyde; NO, nitric oxide; SOD, superoxide dismutase; PR, pulse rate; CFI, cardiac function index; CAT, catalase; CK, creatine kinase; PP, pulse pressure difference; MAP, mean arterial pressure; SVR, systemic vascular resistance; AHAD, acute high altitude disease; HAAI, high altitude adaptation index; bFGF, basic fibroblast growth factor; Hb, haemoglobin; H_2_O_2_, hydrogen peroxide.

### 3.5 Adverse events

Three studies (Cai et al., 2003; He, 2011; Chiu et al., 2013) reported adverse events occurring during the treatment process. In the treatment group, six out of 125 participants (4.8%) experienced adverse events, with the primary adverse events being dizziness and drowsiness. In the control group, 14 out of 131 participants (10.7%) experienced adverse events, with the primary adverse events identified being headache, xerostomia, and gastrointestinal reactions. All of which were mild and self-limiting in nature, with no reports of severe cases. The findings demonstrated that the incidence of adverse events in the experimental group was significantly lower compared to the control group.

### 3.6 Sensitivity analyses

The sensitivity analyses indicated that the results of the meta-analysis remained consistent regardless of the exclusion of any individual study, thereby suggesting the robustness of the findings. Detailed data pertaining to the sensitivity analyses are presented in Supplementary Materia S5.

### 3.7 Subgroup analysis

Subgroup analyses for both primary and secondary outcomes were carried out depending on the treatment durations (≤7 days, >7 days, and no reports) within the same intervention comparisons (RCE vs. WM, RCE vs. placebo) (Table 4; Table 5). In the RCE vs. WM group, the meta-analysis showed no significant difference in total clinical efficacy between the groups for treatment durations of ≤7 days, but RCE is significantly more effective for durations >7 days; the other outcomes showed no statistical significance due to the lack of comparison of treatment durations. In the RCE vs. placebo group, the meta-analysis demonstrated that RCE showed significant efficacy in improving SaO_2_ and PaO_2_, regardless of whether the treatment lasted ≤7 days or >7 days; for reducing SBP and DBP, no statistically significant differences were observed between groups for treatment durations of ≤7 days, but RCE showed significant efficacy for durations >7 days; and regarding HR reduction, there were no statistically significant differences between the two groups for treatment durations of ≤7 days and >7 days; the total clinical efficacy showed no statistical significance due to the lack of comparison of treatment durations.

**TABLE 4:** Subgroup analysis of RCE vs. WM group based on the treatment durations.

Outcome	Subgroup	Number of study	Sample size (T/C)	Measures	Effect estimate (95 %CI)	Heterogeneity (*I* ^*2*^)	*P*_interaction_
SaO_2_	≤7d	1	60/60	MD (N/A)	1.48 [-1.20, 4.16]	-	0.28
	No reports	1	9/13	MD (N/A)	−6.00 [-10.61, −1.39]	-	0.01
PaO_2_	≤7d	1	60/60	MD (N/A)	1.92 [-0.38, 4.22]	-	0.10
Total clinical efficacy	≤7d	2	86/86	RR (Random)	1.12 [0.73, 1.71]	96%	0.61
	>7d	1	15/17	RR (N/A)	1.94 [1.04, 3.62]	-	0.04
SBP	≤7d	1	60/60	MD (N/A)	9.46 [5.58, 13.34]	-	<0.00001
DBP	≤7d	1	60/60	MD (N/A)	3.49 [0.53, 6.45]	-	0.02
HR	No reports	1	9/13	MD (N/A)	6.60 [-2.37, 15.57]	-	0.15

Abbreviations: MD, mean difference; RR, relative risk; CI, confidence interval; *P*_interaction_, *P* for interaction; T, treatment group; C, control group; SaO_2_, arterial oxygen saturation; PaO_2_, arterial partial pressure of oxygen; SBP, systolic blood pressure; DBP, diastolic blood pressure; HR, heart rate.

**TABLE 5:** Subgroup analysis of RCE vs. placebo group based on the treatment durations.

Outcome	Subgroup	Number of study	Sample size (T/C)	Measures	Effect estimate (95 %CI)	Heterogeneity (*I* ^*2*^)	*P*_interaction_
SaO_2_	≤7d	1	37/37	MD (N/A)	11.10 [8.25, 13.95]	-	<0.00001
	>7d	1	110/110	MD (N/A)	3.40 [2.84, 3.96]	-	<0.00001
	No reports	1	9/19	MD (N/A)	7.20 [2.54, 11.86]	-	0.002
PaO_2_	≤7d	1	37/37	MD (N/A)	2.06 [1.43, 2.69]	-	<0.00001
	>7d	1	20/20	MD (N/A)	4.10 [1.37, 6.83]	-	0.003
Total clinical efficacy	≤7d	1	37/37	RR (N/A)	1.42 [1.14, 1.75]	-	0.001
SBP	≤7d	2	124/124	MD (Random)	−2.26 [-6.36, 1.85]	85%	0.28
	>7d	1	50/50	MD (N/A)	−9.40 [-13.59, −5.21]	-	<0.0001
DBP	≤7d	2	124/124	MD (Fixed)	−0.03 [-1.26, 1.20]	0%	0.96
	>7d	1	50/50	MD (N/A)	−4.98 [-8.74, −1.22]	-	0.009
HR	≤7d	2	61/61	MD (Random)	−3.26 [-7.96, 1.44]	83%	0.17
	>7d	1	50/50	MD (N/A)	−2.60 [-8.55, 3.35]	-	0.39
	No reports	1	9/19	MD (N/A)	−0.10 [-9.77, 9.57]	-	0.98

Abbreviations: MD, mean difference; RR, relative risk; CI, confidence interval; *P*_interaction_, *P* for interaction; T, treatment group; C, control group; SaO_2_, arterial oxygen saturation; PaO_2_, arterial partial pressure of oxygen; SBP, systolic blood pressure; DBP, diastolic blood pressure; HR, heart rate.

### 3.8 Publication bias

Due to the limited sample size of the included studies, an assessment of publication bias was not performed.

### 3.9 Certainty of evidence

The certainty of evidence regarding the outcomes was evaluated with the GRADE methodology, as detailed in Table 6. The results demonstrated that the evidences for adverse events were considered to be of moderate certainty. In contrast, the evidences for DBP and HR were assessed as having low certainty, while the evidences for SaO_2_, PaO_2_, SBP and total clinical efficacy were deemed to have very low certainty. The downgrade was primarily due to uncertainty associated with bias and inconsistency.

**TABLE 6:** Certainty of evidence.

Outcome	Quality assessment							No. of patients		Effect		Certainty
	No. of studies	Design	Risk of bias	Inconsistency	Indirectness	Imprecision	Publication bias	T	C	Relative (95% CI)	Absolute (95% CI)	
SaO_2_	5	RCT	serious	very serious	not serious	not serious	N/A	225	239	N/A	MD 3.27 (−0.73–7.28)	⊕ΟΟΟ VERY LOW
PaO_2_	3	RCT	serious	very serious	not serious	not serious	N/A	117	117	N/A	MD 1.93 (0.2–3.65)	⊕ΟΟΟ VERY LOW
Total clinical efficacy	5	RCT	serious	very serious	not serious	not serious	N/A	133/138 (96.4%)	108/140 (77.1%)	RR 1.32 (0.84–2.08)	N/A	⊕ΟΟΟ VERY LOW
SBP	4	RCT	serious	very serious	not serious	not serious	N/A	234	234	N/A	MD 1.14 lower (7.22 lower to 4.94 higher)	⊕ΟΟΟ VERY LOW
DBP	4	RCT	serious	serious	not serious	not serious	N/A	234	234	N/A	MD 0.12 lower (2.55 lower to 2.3 higher)	⊕⊕ΟΟ LOW
HR	5	RCT	very serious	not serious	not serious	not serious	N/A	129	143	N/A	MD 1.9 lower (5.69 lower to 1.9 higher)	⊕⊕ΟΟ LOW
Adverse event	1	RCT	serious	not serious	not serious	not serious	N/A	6/125 (4.8%)	14/131 (10.7%)	RR 0.48 (0.20–1.16)	N/A	⊕⊕⊕Ο MODERATE

Abbreviations: MD, mean difference; RR, relative risk; CI, confidence interval; *P*_interaction_, *P* for interaction; SaO_2_, arterial oxygen saturation; PaO_2_, arterial partial pressure of oxygen; SBP, systolic blood pressure; DBP, diastolic blood pressure; HR, heart rate.

## 4 Discussion

### 4.1 Efficacy of RCE for AHAD

In the context of AHAD, hypoxia represents the predominant pathological mechanism and the most frequently observed clinical symptom, while SaO_2_ and PaO_2_ are the main indicators reflecting the oxygen content in the human body (Wu, 2014). The results indicated that RCE was significantly more effective than the placebo and may be as effective as WM in improving SaO_2_ and PaO_2_ levels. This suggested that RCE may improve blood oxygen levels in patients with AHAD. The primary mechanisms are linked to salidroside, the principal constituent of RCE, which decreases oxygen consumption, scavenges free radicals, mitigates lipid peroxidation reactions, and prevents hemorheological changes induced by hypoxic conditions (Chen et al., 2017; Ma et al., 2019; Hou et al., 2024). It is essential to recognize that the mechanism underlying AHAD cannot be entirely equated with hypoxia. SaO_2_ and PaO_2_ do not fully capture the therapeutic efficacy related to AHAD (Burtscher et al., 2004; Loeppky et al., 2008). Consequently, when citing this article, it is imperative to consider analyses of additional indicators.

AHAD often presents with symptoms like headache, nausea, loss of appetite, digestive issues, insomnia, fatigue, and hair loss due to hypoxia, cold, and radiation (Zhang and Shu, 2011). The meta-analysis demonstrated that RCE exhibited significant efficacy in improving the total clinical efficacy, and it might effectively reduce and shorten high altitude reactions. This efficacy may be closely associated with RCE’s pharmacological properties, including immune regulation, antioxidative activity, anti-inflammatory effects, anti-apoptotic mechanisms, and neuroprotective functions (Yang et al., 2015; Si et al., 2022). However, In terms of reducing SBP and DBP, RCE’s efficacy was inferior to that of WM, and it did not demonstrate significant efficacy relative to placebo. RCE also showed no significant effect in reducing HR. The results indicated that RCE may not have an advantage in acutely reducing elevated SBP, DBP, and HR. This lack of efficacy may be due to its slow action, which fails to counteract the quick damage caused by hypoxia. Its complex makeup, poor specificity, and low concentration of active ingredients also likely reduce its efficacy (Yu et al., 2006; Li et al., 2008). The observed lack of efficacy might also be attributed to confounding variables, including methodological limitations, the formulation type, the route of administration, and the dosage. We also recommend strengthening the comparison with WM, highlighting any potential synergies in combined use.

High-altitude conditions cause various pathophysiological changes in cardiac, pulmonary, and cerebral tissues due to hypoxia acclimatization (Liu et al., 2016). This environment also disrupts oxidative stress balance, resulting in altered blood gas and biochemical parameters (Yang et al., 2011). The results of the meta-analysis demonstrated that RCE was effective in enhancing pulmonary function, cardiovascular function, blood gas analysis, biochemical indices and evaluation index of altitude disease. Previous studies have also shown that RCE may enhance blood oxygen and hemodynamics to boost heart function, lower pulmonary artery pressure and ease vascular tension, thereby preventing and treating AHAD (Yang et al., 2015; Liu et al., 2016). However, its efficacy in improving indicators of brain function did not reach statistical significance.

### 4.2 Safety of RCE for AHAD

According to the results, the occurrence of adverse events was infrequent and of mild severity during the treatment of AHAD with RCE. These results suggested that incorporating RCE into the treatment regimen does not appear to elevate the incidence of additional safety events, thereby implying that RCE may have a favorable safety profile. Nevertheless, given the limited number of studies reporting adverse events that were included in the analysis, this conclusion should be interpreted with caution. Simultaneously, the inconsistencies in standardized reporting of adverse reactions in RCTs may contribute to underreporting. We suggested that future research adhere to international guidelines, such as CONSORT-Harms, to enhance the rigor of safety evaluations. Previous research indicated that RCE may be a contributing factor to adverse events, including rashes, headaches, dizziness, palpitations, nausea, vomiting, anaphylactoid reactions, dyspnea, among others (Wang et al., 2019). Adverse events associated with RCE affect multiple physiological systems, with systemic damage representing the highest incidence, predominantly occurring in elderly individuals (Li et al., 2015). The incidence of these adverse events may be attributed to drug interactions and delayed metabolism in the elderly population. Consequently, it is imperative to exercise caution when administering drug combinations, and particular vigilance should be applied when treating patients with hepatic or renal insufficiency (Li et al., 2015). The adverse reactions associated with RCE continue to be a subject of debate, necessitating further research to elucidate this issue. Additionally, reinforce that use in elderly individuals or those with comorbidities requires special monitoring, especially given altered hepatic and renal metabolism.

### 4.3 Risk of bias

Despite our efforts to mitigate bias throughout the research process, certain factors proved to be unavoidable. Most studies exhibited bias in the randomization process, primarily due to the randomization methodology and the concealment of allocation. Over half of the studies exhibited bias in deviations from the intended interventions, attributable to the absence of blinding. More than 30% of the studies exhibited bias in measurement of the outcome, primarily due to improper measurement methods. All studies demonstrated selection bias in the reported results, primarily due to the absence of a predetermined plan. Furthermore, the lack of intention-to-treat analysis in these studies may lead to an overestimation of the efficacy of RCE. Consequently, the results of this study warrant careful interpretation. This highlighted the critical importance of employing instruments such as the Cochrane Risk of Bias 2.0 (RoB 2.0) in forthcoming research assessments. Moreover, we recommend the prospective registration of studies in databases such as ClinicalTrials.gov or ChiCTR to reduce selection and reporting bias.

### 4.4 Certainty of evidence

The GRADE system was used to assess the certainty of evidence for both primary and secondary outcomes, as well as adverse events. The low certainty of most results made us cautious about the results, primarily due to bias risk and inconsistency. Firstly, the study found that among the overall risks of bias for all outcomes, more than two-thirds were rated as issues of concern. Consequently, the certainty of all outcomes was downgraded by one level. Secondly, in terms of inconsistency in evidence, the heterogeneity test of four outcomes showed that *I* ^*2*^ exceeding 75%, and the evidence was downgraded by two levels. And due to the heterogeneity test of two outcomes showing I^2^ > 50% and <75%, the evidence was downgraded by one level. Due to downgrading, the certainty of the results in this study is affected, and therefore the results should be interpreted with caution. Conducting high-quality RCTs is crucial to enhance the reliability of evidence related to RCE in AHAD. We recommend that future studies increase sample size and standardize clinical outcomes, as heterogeneity may have contributed to the low confidence in the evidence.

### 4.5 Heterogeneity between the included studies

Addressing clinical heterogeneity, this review implemented strict eligibility criteria regarding participants, interventions, comparisons, outcomes, and study designs. Furthermore, the study performed a sensitivity analysis and a subgroup analysis stratified by the treatment duration. However, the clinical heterogeneity observed in some of the results remained inadequately explained, possibly due to the following two factors. Firstly, there were demographic differences among study participants, but age, gender, and comorbidity details were hard to differentiate. Secondly, the intervention measures employed for the control groups in this study comprised WM or placebo. Although the analyses were conducted separately for each, variations in the implementation of WM and placebo were observed across different studies. Future studies must implement stricter control over interventions in the control group, such as clearly defining western medicine treatments or placebo protocols. We also recommend multicenter studies to minimize population heterogeneity, including factors like gender, age, ethnicity, and local altitude.

### 4.6 Clinical implications

The results of this study indicated that RCE may substantially improve hypoxia resulting from high-altitude environments, enhance organ function and optimize physiological and biochemical parameters, and demonstrated favorable safety. Consequently, RCE exhibited potential as a clinical therapeutic agent for the prevention of altitude sickness and the mitigation of symptoms associated with AHAD. It also illustrated the potential applicability of Rhodiola crenulata extract (RCE) as an adjunct to standard treatment in areas with limited access to western medicine. Moreover, the subgroup analysis revealed that RCE’s therapeutic efficacy on AHAD was notably improved when the treatment duration surpassed 7 days, suggesting that a longer treatment course might result in better efficacy of RCE in the treatment of AHAD. This suggested that the treatment duration (exceeding 7 days) may be a key variable for efficacy, which may have implications for prophylactic use logistics before travel to high altitudes.

### 4.7 Strengths and limitations

This study constituted a pioneering systematic review and meta-analysis examining the efficacy of RCE in the treatment of AHAD. We meticulously implemented stringent inclusion and exclusion criteria to mitigate the confounding effects of other traditional Chinese medicine interventions. Our analysis incorporated data from 1,690 participants across 19 randomized controlled trials, yielding a substantial sample size that provides compelling clinical evidence regarding the effectiveness of RCE in the management of AHAD. Additionally, the influence of treatment duration on the efficacy of RCE in the management of AHAD was explored through a subgroup analysis.

Nevertheless, this review was subject to several limitations. First, the low quality of the included studies undermined the credibility of the research findings. Second, all 19 RCTs included in this study were conducted exclusively in China, with no representation from other countries. Third, this analysis was influenced to several confounding variables, such as the inconsistent dosages, administration frequency and treatment durations of RCE reported in the original studies, the omission of participants’ prior experience with herbal medicine, and the lack of documentation regarding the altitudes at the journey’s commencement and destination. These factors may compromise the accuracy of the analysis. Fourth, notwithstanding the implementation of sensitivity and subgroup analyses, the meta-analysis results for both primary and secondary outcomes exhibited significant heterogeneity, the origins of which were difficult to determine. Fifth, the absence of follow-up time reported in the studies hinders the assessment of RCE’s long-term prognosis in patients.

### 4.8 Future perspectives

We recommend broader geographic representation in future studies (beyond China). To enhance the quality of literature, it is recommended that future studies will employ more robust research designs, reinforce quality control throughout the research implementation process, and undertake multi-center, large-sample, double-blind RCTs, and include international research. In the design of clinical trials, it is advisable to incorporate functional and quality-of-life outcomes alongside laboratory indicators. Additionally, metrics for evaluating long-term efficacy, such as recurrence rates and mortality, should be included.

Furthermore, the pharmacological mechanism through which RCE exerts its therapeutic effects in the treatment of AHAD requires further investigation. It is recommended that future studies explore the specific molecular pathways of RCE in both animal and human models, with a particular focus on its antioxidant, anti-apoptotic, and vascular mechanisms. Employing preclinical AMS models in conjunction with neuroinflammatory or mitochondrial biomarkers could significantly enhance the understanding of RCE’s mechanisms. Additionally, there is a need to develop standardized RCE formulations with rigorous quality control of active compounds to ensure consistency and efficacy in research and therapeutic applications.

## 5 Conclusion

This systematic review and meta-analysis suggesed that *R. crenulata* extract may offer therapeutic potential for Acute high altitude disease, particularly by improving blood oxygenation and alleviating clinical symptoms, with a favorable safety profile. Notably, prolonged use appears to enhance its efficacy. However, the overall certainty of the evidence remained low due to methodological limitations in the included studies. Therefore, there is an urgent need for robust, well-designed, multicenter randomized clinical trials to validate these findings and clarify the long-term safety and effectiveness of RCE in managing AHAD.

## Data Availability

The original contributions presented in the study are included in the article/Supplementary Material, further inquiries can be directed to the corresponding authors.

## Abbreviations

AHAD, acute high-altitude disease; PaO_2_, arterial partial pressure of oxygen; CAT, catalase; PASP, pulmonary arterial systolic pressure; CFI, cardiac function index; PP, pulse pressure difference; CI, confidence interval; PRISMA, 2020, Preferred Reporting Items for Systematic Reviews and Meta-Analyses 2020; CK, creatine kinase; RCE, *R. crenulata* extract; CNKI, China National Knowledge Infrastructure Database; RCT, randomized controlled trial; DBP, diastolic blood pressure; ROB 2.0) the revised Cochrane risk of bias tool for randomized trials 2.0; GRADE, Grading of Recommendations Assessment, Development and Evaluation; RR, relative risk; HAAI, high altitude adaptation index; SaO_2_, oxygen saturation; AHAD, high altitude diseases; SBP, systolic blood pressure; Hb, haemoglobin; SinoMed, Chinese Biomedical Literature Database; H_2_O_2_, hydrogen peroxide; TCM, Traditional Chinese Medicine; HR, heart rate; VEI, ventilation efficiency index; MD, mean difference; VIP, VIP Database for Chinese Technical Periodicals; MDA, malondialdehyde; WF, Wanfang Database; MPAP, mean pulmonary arterial pressure; WM, western medicine; NO, nitric oxide; PaCO_2_, arterial partial pressure of carbon dioxide.
