# Supplementary material for: Efficacy and safety of Rhodiola crenulata extract in the treatment of acute high altitude disease, based on studies involving populations in China: A systematic review and meta-analysis
Source: Front Pharmacol. 2025 Jun 13;16:1595953. doi: 10.3389/fphar.2025.1595953 (PMC12202619; doi:10.3389/fphar.2025.1595953)
Supplement: Supplementary file 1 [file DataSheet1.pdf]

Supplementary Material

SI The Nomenclature, Classification and Diagnostic Criteria of High Altitude Disease

1. Nomenclature and Classification

1.1 Acute High Altitude Disease (AHAD)（AMS）

(1) Mild type

1) Acute Mild Altitude Disease (AMAD)

(2) Serious type

1) High Altitude Pulmonary Edema (HAPE)

2) High Altitude Cerebral Edema (HACE)

2. Clinical Diagnostic Criteria of High Altitude Disease (HAD)

2.1 Diagnostic criteria and scoring system of acute mild high altitude disease (AMAD)

Table 1 shows the questionnaire scoring system used to classify the disease based on the severity of symptoms. Table 2 shows the degree and grade based on the severity of the disease.

Supplementary Table 1 Assessment of the Degree and Score of AMAD

Symptoms	Degree	Score
**Headache**		
1. No headache, no suffering expression, no effect on daily activity	±	1
2. Mild headache with suffering expression; obvious improvement of headache after taking regular analgesic medicine; no effect on daily activity.	+	2
3. Moderate headache with suffering expression; slight improvement of headache after taking regular analgesic medicine; daily activity is affected.	++	4
4. Severe and unbearable headache; lie in bed and cannot get up; no effect of regular analgesic medication.	+++	7
**Vomiting**		
1. Vomiting 1-2 times a day; vomit contains only intaken food, obvious improvement with regular anti-vomit medication; no effect on daily activity.	+	2
2. Vomiting 3-4 times a day; final vomit contains gastric juice; slight improvement with anti-vomit medication; daily activity is affected.	++	4
3. Vomiting more than 5 times a day; must lie in bed and cannot get up, no improvement with regular anti-vomit medication.	+++	7
**Other**		
Dizziness/lightheadedness, nausea, palpitation, short of breath, chest		1 point

distress, dazzling/blurred vision, sleeplessness (insomnia), anorexia, abdominal distension, diarhea, constipation, cyanosis of the lips, lethargy, and numbness of the extremities	each

Supplementary Table 2 Assessment of the Severity and Grade of AMHAD

Degree	Score/Grade
Normal(±)	Total score of 1-4 points
Mild(+)	Headache +, or vomiting +; or total score of 5-10 points
Moderate(++)	Headache ++,or vomiting ++; or total score of 11-16 points
Severe(+++)	Headache +++, or vomiting +++; or total score of 16 points

2.2 Diagnostic criteria of high altitude pulmonary edema(HAPE)

2.2.1 Field (or on site) diagnosis

(1) Cause of the disease: Recent arrival at high altitude area (A recent gain in high altitude)(generally >3,000 m above sea level).

(2) Symptoms: dyspnea at rest, chest distress, cough with white or pinkish froth sputum, fatigue, asthenia or decreased capacity of activities.

(3) Signs: moist rales or wheezing in one or both lung fields, central cyanosis, tachycardia, and tachypnea.

The final diagnosis of HAPE should include at least two of the symptoms and signs mentioned above

2.2.2 Clinical diagnostic criteria

(1) Recent arrival at high altitude area (A recent gain in high altitude) (generally > 3,000 m above sea level), dyspnea at rest, cough with white or pinkish froth sputum.

(2) Central cyanosis, pulmonary moist rales.

(3) Chest X-ray is the major criteria for diagnosis. Patients often have patchy or "cloudy"infiltrate shadow centered on the hila and radiated to one or two sides of lung fields. The opacification in the lung is often diffused and irregular, but can also be diffused into an enlarged shadow. Cardiac imagine is often normal, but can show changes indicative of pulmonary hypertension and right heart enlargement.

(4) The diagnosis requires exclusion of acute myocardial infarction, acute cardiac failure due to heart disease, pneumonia and other cardiopulmonary diseases on the basis of clinical examination and ECG.

(5) Prompt improvement of the signs and symptoms after resting in bed and oxygen therapy, or after descending to low altitude. Disappearance of X-ray abnormalities within a short period of time.

2.3 Diagnostic criteria of high altitude cerebral edema (HACE)

2.3.1 Field (or on site) diagnosis

(1) Recent arrival at high altitude area (A recent gain in high altitude) (generally >3,000 m above sea level); patients often had severe AMAD prior to HACE.

(2) The presence of mental status changes and/or ataxia in a person with AMAD, or the concurrent presence of mental status changes and ataxia in a person without AMAD (The changes in mental status are graded, according to the severity, into apathy, lethargy or lassitude, disorientation or confusion, stupor or semiconscious, and coma. The severity of ataxia can be determined on the basis of the following signs and symptoms: loss control of balance, unable to walk straight or step off line, falling, and unable to stand).

2.3.2 Clinical diagnostic criteria

(1) Recent arrival at high altitude area (A recent gain in high altitude) (generally>3000 m above sea level).

(2) Neurological and mental (psychological) symptoms: severe headache, vomiting, apathy, mental depression or euphoria and polylogia, dysphoria, staggering (or reeling) gait, ataxia (Romberg sign positive). It is then followed by mental confusion, haziness, drowsiness or somnolence, and coma; or become coma directly. Physical incapacity, the signs and symptoms due to meningeal irritation and/or positive pyramidal sign may occur as well.

(3) Ocular fundus: may have papilledema and/or retinal hemorrhage.

(4) Cerebrospinal fluid (CSF): Increased pressure, no change in cells and protein. On rare occasions, blood or red blood cells are present in CSF.

(5) Exclude the possibility of acute cerebral vascular disease, acute toxic effect of medication and acute poisoning of carbon monoxide, epilepsy, meningitis, and central nervous system infection.

(6) Prompt improvement of the signs and symptoms after treatment with oxygen, diuretics, and steroid, or descending to low altitude.

SII Comprehensive information regarding the RCE

Supplementary Table 3 The information of Chinese patent medicine

Chinese patent medicine	Primary therapeutic components	Manufacturer	Efficacy of Chinese patent
Hongjingtian Capsule	*Rhodiola crenulata*	1. Tibet Lhasa Shengya Pharmaceutical Center<br>2. Tibet Military Region Hongjingtian Research and Development Center of the Chinese People's Liberation Army<br>3. Information Institute of Traditional Chinese Medicine, China Academy of Chinese Medical Sciences<br>4. Tibet Yangke	Enhancing blood circulation to alleviate blood stasis and facilitating the unblocking of meridians to relieve pain.

		Biotechnology Company<br>5. Jiangsu Kanion Pharmaceutical Co., Ltd.	
Hongjingtian Tablet	*Rhodiola crenulata*	1. Sichuan Tiansheng Pharmaceutical Co., Ltd.<br>2. Jiangsu Kanion Pharmaceutical Co., Ltd.	Enhancing blood circulation to alleviate blood stasis and facilitating the unblocking of meridians to relieve pain.
Hongjingtian Oral Solution	*Rhodiola crenulata*	1. Tibet Tibetan Medicine Group Co., Ltd.<br>2. Hangzhou Huawei Pharmaceutical Co., Ltd.<br>3. Tibet Yakang Natural Pharmaceutical Co., Ltd.	Facilitating the circulation of Qi (vital energy) and blood.
Nuodikang Capsule	*Rhodiola crenulata*	Tibet Nuodikang Pharmaceutical Co., Ltd.	Facilitating the circulation of Qi (vital energy) and blood, as well as unblocking meridians, to alleviate pain.
Nuodikang Tablet	*Rhodiola crenulata*	Sichuan Nuodikang Weiguang Pharmaceutical Co., Ltd.	Facilitating the circulation of Qi (vital energy) and blood.
Nuodikang Granule	*Rhodiola crenulata*	Tibet Nuodikang Pharmaceutical Co., Ltd.	Facilitating the circulation of Qi (vital energy) and blood, as well as unblocking meridians, to alleviate pain.
Nuodikang Oral Solution	*Rhodiola crenulata*	Chengdu Nuodikang Biopharmaceutical Co., Ltd.	Facilitating the circulation of Qi (vital energy) and blood, as well as unblocking meridians, to alleviate pain.

SIII Search strategies

The search queries for China National Knowledge Infrastructure Database (CNKI), VIP Database for Chinese Technical Periodicals (VIP), Wanfang Database (Wanfang), Chinese Biomedical Literature Database (SinoMed), Web of Science, Pubmed, Embase, and Cochrane Library are shown in Table 1.

Supplementary Table 4 Search strategies for each database

Database	Search strategies
CNKI	(TKA = '高原病' OR TKA = '高原反应' OR TKA = '高原') AND (TKA = '红景天' OR TKA = '红景天制剂') AND FT = '随机'
VIP	M = (高原病 OR 高原反应 OR 高原) AND M = (红景天 OR 红景天制剂) AND U = 随机
Wanfang	主题: (高原病 or 高原反应 or 高原) and 主题: (红景天 or 红景天制剂) and 全部: (随机)
SinoMed	("随机"[全部字段:智能]) AND ("红景天"[常用字段:智能] OR "红景天制剂"[常用字段:智能]) AND ("高原病"[常用字段:智能] OR "高原反应"[常用字段:智能] OR "高原"[常用字段:智能])
Web of Science	#1 TS=(Altitude Sickness OR Altitude Diseases OR Sickness, Altitude OR Diseases, Altitude OR Altitude Hypoxia OR Altitude Hypoxias OR Hypoxia, Altitude OR Mountain Sickness OR Sickness, Mountain)<br>#2 TS=(*Rhodiola crenulata* OR *Rhodiola rosea* OR *Roseroot* OR *Roseroots* OR Hongjingtian OR Hong jing tian)<br>#3 (ALL=(randomized) OR TS=(RCT))<br>#4=#1 AND #2 AND #3
Pumbed	#1 "Altitude Sickness" [MeSH Terms] OR "Altitude Diseases" [Title/Abstract] OR "Sickness, Altitude" [Title/Abstract] OR "Diseases, Altitude" [Title/Abstract] OR "Altitude Hypoxia" [Title/Abstract] OR "Altitude Hypoxias" [Title/Abstract] OR "Hypoxia, Altitude" [Title/Abstract] OR "Mountain Sickness" [Title/Abstract] OR "Sickness, Mountain" [Title/Abstract]<br>#2 "*Rhodiola crenulata*" [MeSH Terms] OR "*Rhodiola rosea*" [Title/Abstract] OR "*Roseroot*" [Title/Abstract] OR "*Roseroots*" [Title/Abstract] OR "Hongjingtian" [Title/Abstract] OR "Hong jing tian" [Title/Abstract]<br>#3 "randomized controlled trial" [Publication type] OR "randomized clinical trial" [Publication type] OR "randomized trial" [Publication type] OR "clinical trial" [Publication type] OR "randomized controlled trial" [Title/Abstract] OR "randomized clinical trial" [Title/Abstract] OR "randomized trial" [Title/Abstract] OR "clinical trial" [Title/Abstract]<br>#4 #1 AND #2 AND #3
Embase	#1 'altitude diseases':ti,ab,kw OR 'sickness, altitude':ti,ab,kw OR 'diseases, altitude':ti,ab,kw OR 'altitude hypoxia':ti,ab,kw OR 'altitude hypoxiase':ti,ab,kw OR 'hypoxia, altitude':ti,ab,kw OR 'mountain sickness':ti,ab,kw OR 'sickness, mountain':ti,ab,kw<br>#2 'altitude sickness'/exp<br>#3=#1 OR #2<br>#4 '*Rhodiola crenulata*'/exp<br>#5 '*rhodiola rosea*':ti,ab,kw OR *roseroot*:ti,ab,kw OR *roseroots*:ti,ab,kw OR hongjingtian:ti,ab,kw OR hong jing tian:ti,ab,kw<br>#6= #4 OR #5

	#7 'randomized controlled trial'/exp
	#8 ' randomized clinical trial':ti,ab,kw OR 'randomized trial':ti,ab,kw OR 'clinical trial':ti,ab,kw
	#9=#7 OR #8
	#10=#3 AND #6 AND #9
Cochrane Library	#1 MeSH descriptor: [Altitude Sickness] explode all trees
	#2 (Altitude Diseases OR Sickness, Altitude OR Diseases, Altitude OR Altitude Hypoxia OR Altitude Hypoxias OR Hypoxia, Altitude OR Mountain Sickness OR Sickness, Mountain):ti,ab,kw
	#3=#1 OR #2
	#4 MeSH descriptor: [*Rhodiola crenulata*] explode all trees
	#5 (*Rhodiola rosea* OR *Roseroot* OR *Roseroots* OR Hongjingtian OR Hong jing tian):ti,ab,kw
	#6=#4 OR #5
	#7 MeSH descriptor: [Randomized Controlled Trial] explode all trees
	#8 (randomized controlled trial OR randomized clinical trial OR randomized trial OR clinical trial):ti,ab,kw
	#9=#7 OR #8
	#10=#3 AND #6 AND #9

SIV Results of the sensitivity analysis

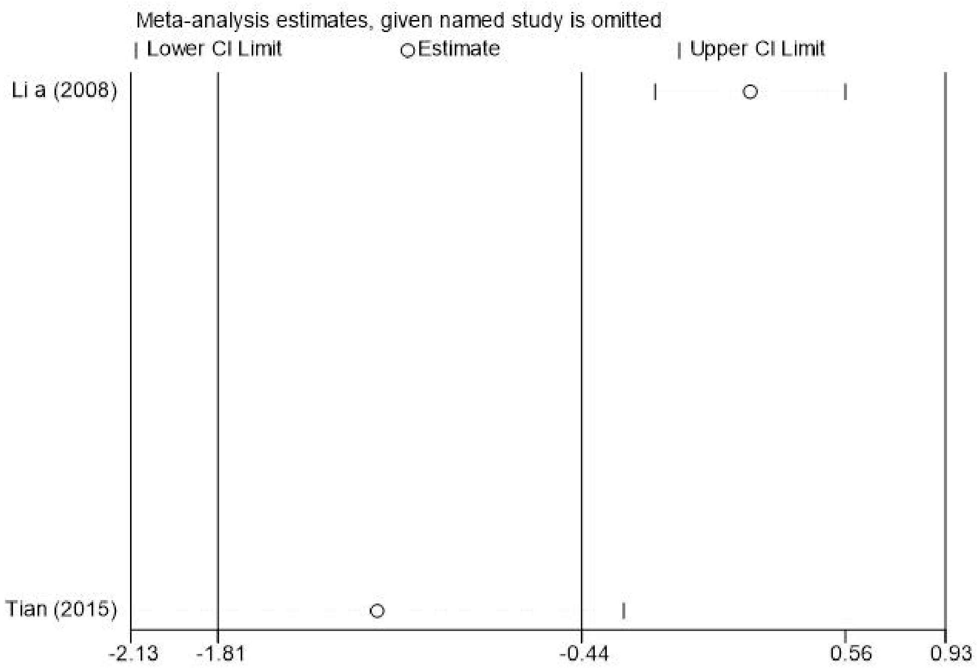

Supplementary Figure 1. The results of the sensitivity analysis of SaO_2 (RCE vs WM). RCE, *Rhodiola crenulata* extract; WM, western medicine.

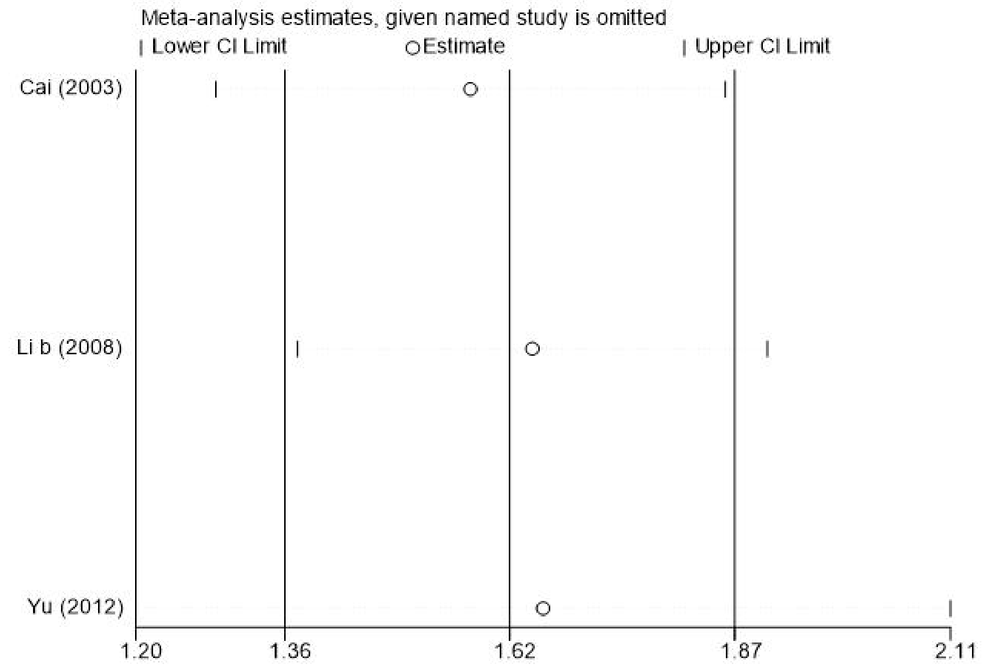

Supplementary Figure 2. The results of the sensitivity analysis of SaO_2 (RCE vs placebo). RCE, *Rhodiola crenulata* extract.

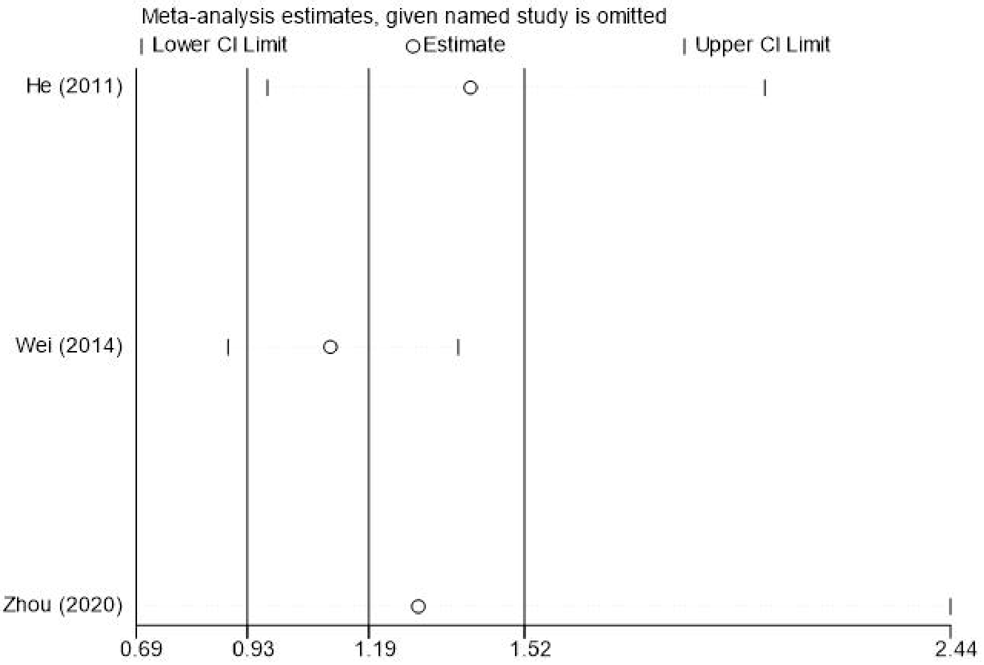

Supplementary Figure 3. The results of the sensitivity analysis of total clinical efficacy (RCE vs WM). RCE, *Rhodiola crenulata* extract; WM, western medicine.

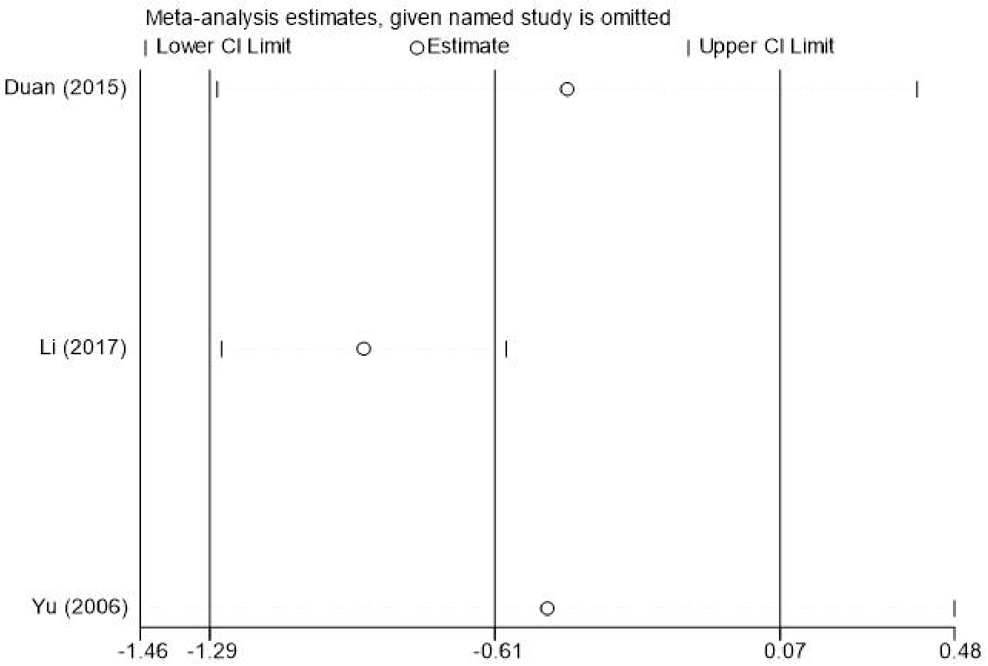

Supplementary Figure 4. The results of the sensitivity analysis of SBP (RCE vs placebo). RCE, *Rhodiola crenulata* extrac.

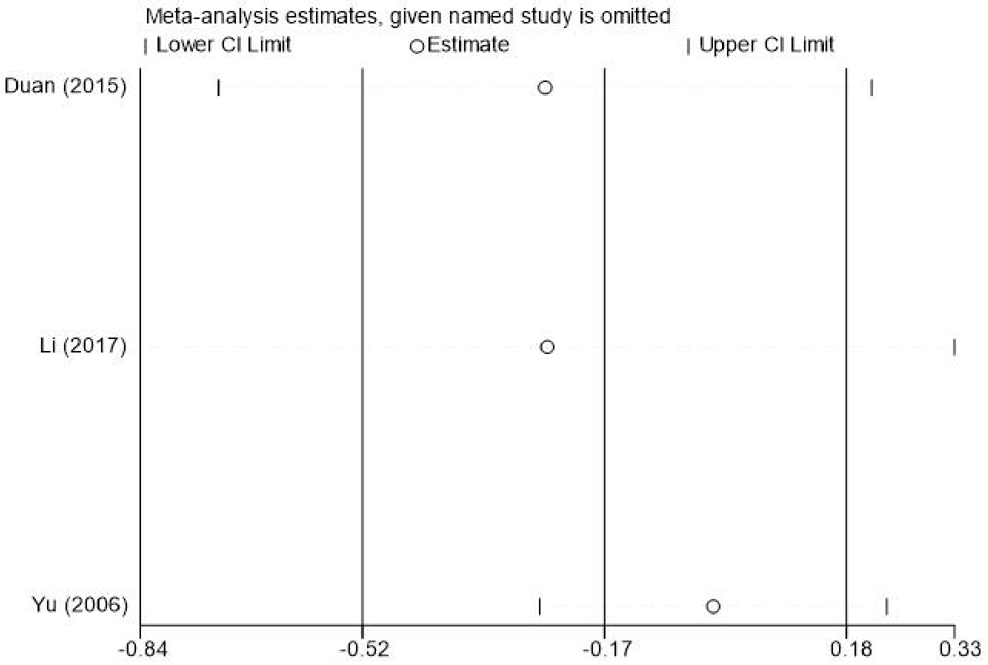

Supplementary Figure 5. The results of the sensitivity analysis of DBP (RCE vs placebo). RCE, *Rhodiola crenulata* extract.

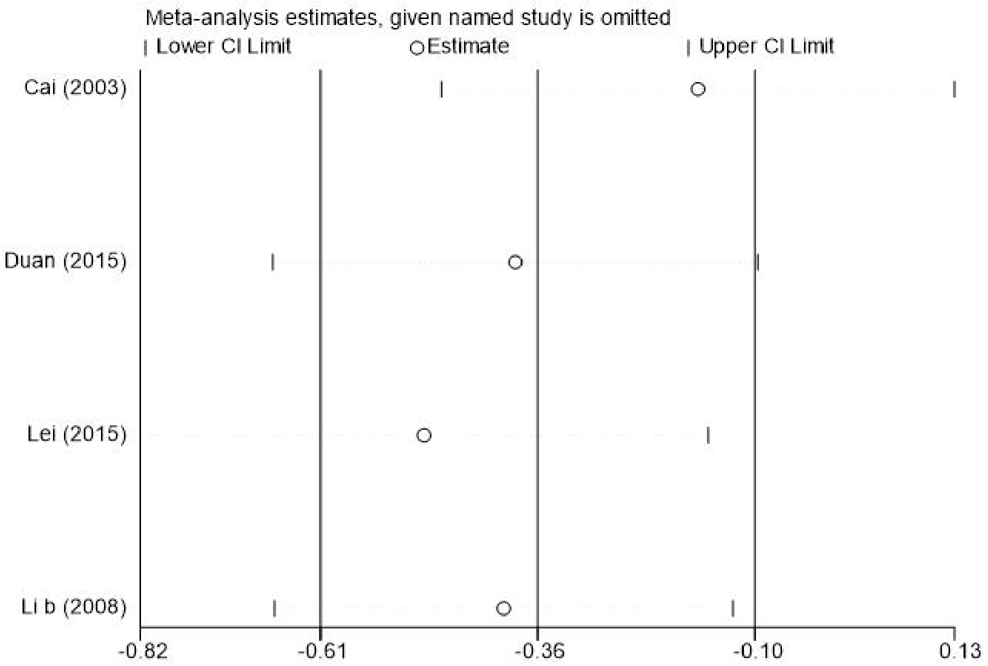

Supplementary Figure 6. The results of the sensitivity analysis of HR (RCE vs placebo). RCE, *Rhodiola crenulata* extract.
